# Estimating excess mortalities due to the COVID-19 pandemic in Malaysia between January 2020 and September 2021

**DOI:** 10.1038/s41598-022-26927-z

**Published:** 2023-01-03

**Authors:** Vivek Jason Jayaraj, Diane Woei-Quan Chong, Kim-Sui Wan, Noran Naqiah Hairi, Nirmala Bhoo-Pathy, Sanjay Rampal, Chiu-Wan Ng

**Affiliations:** 1 Department of Social and Preventive Medicine, Centre for Epidemiology and Evidence-Based Practice, Faculty of Medicine, University of Malaya, 50603 Kuala Lumpur, Malaysia; 2 Ministry of Health Malaysia, Federal Government Administrative Centre, 62590 Putrajaya, Malaysia

**Keywords:** Viral infection, Epidemiology

## Abstract

Excess mortalities are a more accurate indicator of true COVID-19 disease burden. This study aims to investigate levels of excess all-cause mortality and their geographic, age and sex distributions between January 2020-September 2021. National mortality data between January 2016 and September 2021 from the Department of Statistics Malaysia was utilised. Baseline mortality was estimated using the Farrington algorithm and data between 1 January 2016 and 31 December 2019. The occurrence of excess all-cause mortality by geographic-, age- and sex-stratum was examined from 1 January 2020 to 30 September 2021. A sub-analysis was also conducted for road-traffic accidents, ethnicity and nationality. Malaysia had a 5.5–23.7% reduction in all-cause mortality across 2020. A reversal is observed in 2021, with an excess of 13.0–24.0%. Excess mortality density is highest between July and September 2021. All states and sexes reported excess trends consistent with the national trends. There were reductions in all all-cause mortalities in individuals under the age of 15 (0.4–8.1%) and road traffic accident-related mortalities (36.6–80.5%). These reductions were higher during the first Movement Control Order in 2020. Overall, there appears to be a reduction in all-cause mortality for Malaysia in 2020. This trend is reversed in 2021, with excess mortalities being observed. Surveillance of excess mortalities can allow expedient detection of aberrant events allowing timely health system and public health responses.

## Introduction

The World Health Organization declared COVID-19 a pandemic on 11 March 2020. By 31 December 2021 it had spread to over 237 countries and territories worldwide, resulting in over 286 million confirmed cases and approximately 5.43 million deaths^1^. However, these figures are likely to be an underestimation of the true burden of the pandemic^2^. The underestimation of disease burden has been driven by differentials in the capacity to test, overwhelmed health systems and paradoxical increments of risk due to the implementation of restrictive public health and social measures^3–5^.

Malaysia is an upper-middle-income country with a population of 32.7 million^6^. The first COVID-19 case was detected on 25 January 2020, and the first major outbreak occurred in March 2020, with the epicentre traced to a religious event in the state of Selangor^7^. In response to the transmission of COVID-19, Malaysia employed several public health and social measures to contain the spread of COVID-19. These measures were collectively referred to as the Movement Control Order (MCO), included non-pharmaceutical interventions such as border closures, closures of most economic sectors, restrictions on social gatherings and movement of individuals from their homes. They were first implemented on 18 March 2020, leading to decay in transmission^8^. A total of 5,945 cases and 100 deaths were detected throughout the country between 1 January 2020 and 30 April 2020^8^. As transmission decayed, a phased relaxation of measures was initiated. The Conditional Movement Control Order (CMCO) was implemented on 4 May 2020, and the Recovery Movement Control Order (RMCO) on 9 June 2020.

In early September, a state election was held in Sabah on 26 September 2020, triggering a new outbreak of cases that then spread to the rest of Malaysia^9^. Outbreaks over this period were driven by clusters in factories, prisons, and immigration detention centres^8^. The government continued to calibrate the MCO and CMCOs at the state level in controlling transmission. By the end of 2020, Malaysia had 110,485 cases and 463 deaths^10^. The emergence of more transmissible variants in late 2020 further enhanced transmission over 2021 with a particularly damaging Delta wave in mid-2021^11^. The government reconfigured its response in June 2021. The National Recovery Plan was a four-phase data-driven holistic plan aimed at reducing transmission, protecting lives and livelihoods and enhancing economic recovery^12^. By 31 December 2021, Malaysia had recorded 2.75 million cases (84,235 per million population) and 31,462 deaths (962 per million population)^10^.

Malaysia has a parallel public–private health system. The tax-funded public health system has taken the brunt of efforts to vaccinate, screen and treat the populace since the start of the pandemic. As part of plans to cope with mounting cases, many public hospitals and clinics have been set aside for COVID-19 services, with preparedness beginning as early as January 2020. The role of private providers became more defined only later in the pandemic when they began to provide vaccination services, treat limited numbers of COVID-19 patients and receive non-COVID-19 patients decanted from overwhelmed public hospitals.

Historically all-cause mortality has been used for the surveillance of infectious diseases such as influenza and meteorological phenomena such as heatwaves^13,14^. The tracking of all-cause mortality has come to be accepted as a more accurate measure of disease burden within surveillance systems^15,16^. Detecting aberrant events within signals of all-cause mortality trends can be translated into timely public health interventions^13,17^. These algorithms have also been utilised in detecting excess mortalities^18,19^.

Its use as an extension of current COVID-19 surveillance mechanisms can provide greater granularity of disease severity and possible indirect causes of death, leading to a more efficient response of the health systems towards infection risk within the population. Globally, this has been the case in several countries. Estimates in Italy, for instance, have reported a spike in excess mortalities within the elderly population with a spatial predisposition in its northern regions^20^. Similar findings have been reported in the United States and the United Kingdom^21–23^. Although daily statistics on COVID-19 disease burden are provided, excess mortalities are more reflective of disease burden as it can also capture unreported COVID-19 mortalities and non-COVID-19 deaths that are collateral damage from an overwhelmed health system. As such, we aim to investigate the possibility of excess all-cause mortalities in Malaysia due to the COVID-19 pandemic and its age-, location-, and cause-specific distributions across Malaysia.

## Methods

### Data

The laws in Malaysia require that all deaths in the country be registered with the National Registration Department (NRD) within 7 days of death (for states in the peninsula and Sarawak) and 10 days (for Sabah). Mortality data from all states will then be compiled into a national mortality database maintained by the Department of Statistics Malaysia (DOSM)^24^. This national statistical agency will liaise with the Ministry of Health and the Police Department to counter-check for completeness of death registration. During the early phases of the COVID-19 pandemic in 2020, there were disruptions to services provided by the NRD. Service counters were closed from the start of the MCO in March to May 2020. Since March 2020, the period allowed for registrations of deaths was extended to 90 days. There can be a reporting delay of up to three months before DOSM releases its quarterly updates on mortality statistics.

This analysis was carried out using mortality data obtained from DOSM for deaths occurring between epidemiological week one in January 2016 to epidemiological week 39 in September 2021, amounting to 69 months of data. The data included age at death, sex, nationality, ethnicity, place of residence by state, and cause of death. The DOSM codes cause of death using the International Classification of Diseases Version 10 (ICD-10) for deaths that had been medically certified (certified by a medical practitioner, Coroner or Magistrate) or using less formal description codes for deaths that had been certified by laypersons such as police personnel. ICD-10 codes were unavailable for 2019 and 2021 at data extraction. Deaths from road traffic accidents in 2016, 2017, 2018 and 2020 were identified using ICD-10 codes. Deaths due to road traffic accidents for the years 2019 and 2021 were identified by mining free-text descriptions of cause of death keywords such as “motor”, “traffic”, “collision”, “road”, and “crash”, as well as the Malay equivalent of these terms.

Additionally, data for COVID-19 case and mortality line lists were retrieved from the official Ministry of Health Malaysia COVID-19 data repository between 1 January 2020 and 30 September 2021. The data on this repository is owned by the Ministry of Health Malaysia and is updated daily^10^. COVID-19 mortalities in Malaysia are adjudicated as either death due to COVID-19 or deaths with COVID-19 using a pre-defined set of criteria by the Ministry of Health Malaysia. Only deaths due to COVID-19 are counted as COVID-19 mortalities^25^.

### Statistical analysis

Random imputation of dates was carried out for data with no day of death but with month and year of death. This affected 2% of deaths in 2017, 2018 and 2019. Dates were then utilised to categorise mortalities into epidemiologic weeks. A seasonal and trend decomposition using LOESS (STL) was carried out on the time series to explore the temporal structure of the data (Supplementary Appendix 1)^26^. Surveillance of potential aberrations within all-cause mortality counts was carried out using the flexible Farrington algorithm utilised widely in excess mortality detection of COVID-19^19,27–32^. The algorithm is formulated to predict an observed number of counts ${y}_{{t}_{0}}$ at time point, ${t}_{0}$= (${t}_{0}^{m}, {t}_{0}^{y}$) using a set of reference values from a window of size *w* weeks up to *b* years back. These reference values were fit into an overdispersed Poisson generalised linear model, which is given by;

$$\mathrm{log}{\mu }_{t}=\alpha +\beta t+ \gamma c\left(t\right),$$

$$where, {\mu }_{t}\mathrm{ is \, the \, predicted \, count \, at \, week }t, \alpha ,\mathrm{ and }\beta \mathrm{are \, regression \, parameters \, of \, time}-\mathrm{trend},\mathrm{ and }\gamma c\left(t\right) \mathrm{is \, the \, seasonal \, factor \, of \, week }t$$

The model was used to estimate the expected number of mortalities with a one-sided confidence interval. A *w* of 3 and *b* of 5 were utilised, as reported by previous studies^32^. Predictions have been found to be more stable when more historical data is used^28^. As such, the data was further divided into ten periods and fit into the model incorporating this effect^32^. Sensitivity analysis was performed by varying *w* (2–8) and *b* (2–5). Linear trends were tested over time at the 5% significant level for inclusion within the model. Data from 2016–2019 was utilised for model training. No known events causing a surge of all-cause mortalities were identified within the literature between 2016 and 2019. Variance and low counts were checked. Transformations were utilised if skewness and low counts were found. The point estimates and upper limits of prediction intervals were tabulated and visualised across the time series against observed cases.

The analysis was further stratified to observe effects at different levels of society by location and age groups. Locations were divided by the 13 states and three federal territories in Malaysia. The age groups were categorised as; (i) under one, (ii) under five, (iii) 5–14, (iv) 15–40, (v) 41–59, and (vi) 60 years and above^33^. We further stratified the results by sex—either male or female. Stratifications by cause of death—road traffic accidents (RTA) or non-RTA were conducted to investigate the effects of reductions in population mobility on mortality. Further stratifications by ethnicity and nationality were carried out as a sensitivity analysis. Ethnicities were categorised as: (i) Malay, (ii) Other Indigenous, (iii) Chinese, (iv) Indian, and (v) Other ethnicities. Nationalities were categorised as: (i) Malaysian and (ii) Non-Malaysian. Excess mortalities were reported as the difference and percentage change between observed mortalities, and the predicted point estimate, and its 95% confidence interval (upper limit). Excess mortalities attributable to COVID-19 mortalities were calculated by taking the proportion of COVID-19 mortalities from excess mortalities. A sensitivity analysis was carried out to compare the Farrington algorithm to other more recent approaches. A Bayesian hierarchical approach modelling excess mortalities whilst accounting for population trends, temperature, spatiotemporal patterns and a greater geographical resolution developed by Konstantinoudis et al. was implemented for this purpose^34^. All analysis was carried out using the “*tidyverse*” and “*surveillance*” packages in R version 3.6.0^35–37^.

### Ethics declaration

This study was conducted according to the guidelines of the Declaration of Helsinki and approved by the University of Malaya Research Ethics Committee (UM.TNC2/UMREC, 10 June 2020).

## Results

**Figure 1:**
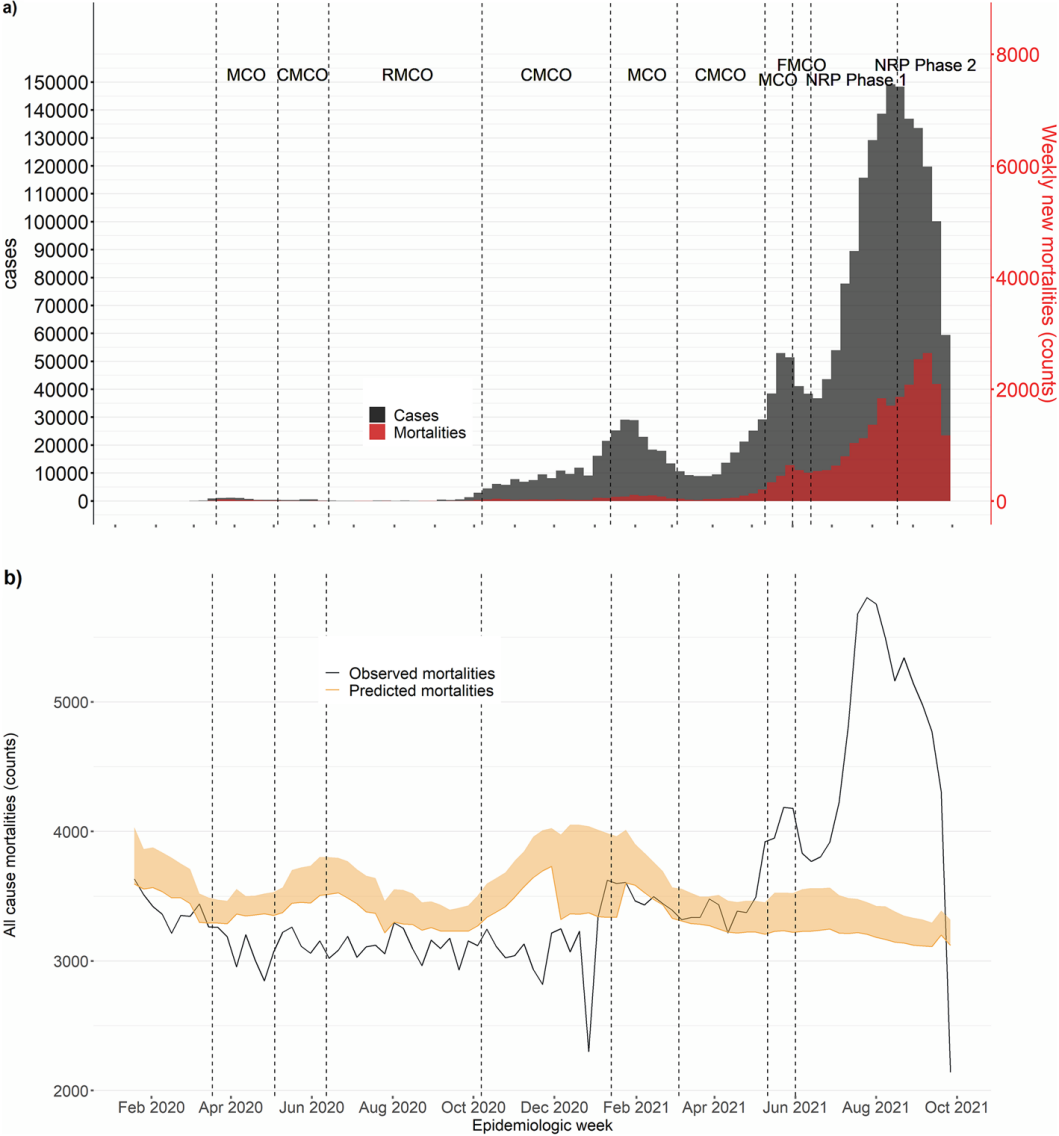
(**a**) Epidemiological curve of COVID-19 cases and deaths over differing phases of interventions in Malaysia; (**b**) Epidemiological curve of COVID-19 mortalities with observed and predicted all-cause mortalities in Malaysia. Note: These policies were for periods calibrated at the state level (indicated below for MCO, NRP calibrated at state level) and the earliest date any state transitioned into a new phase within Malaysia is provided below. Period of implementation: (i) Movement Control Order, MCO, (ii) Conditional Movement Control, CMCO (4 May 2020–8 June 2020), (iii) Recovery Movement Control Order, RMCO (9 June 2020–6 October 2020), (iv) CMCO-targeted by states (7 October 2020–12 January 2021), (v) MCO-targeted by states (13 January 2021–4 March 2021), (vi) CMCO-targeted by states (5 March 2021–10 May 2021), vii) MCO (11 May-31 May 2021), (viii) Full Movement Control Order, FMCO (1 June 2021–14 June 2021), (ix) National Recovery Plan, NRP Phase 1 (15 June 2021–19 August 2021), and (x) NRP Phase 2 (20 August 2021–30 September 2021).

A total of 1,000,562 all-cause mortalities were reported in Malaysia between January 2016 and September 2021. The mean age of all-cause mortalities was 65.6 years old, with 66.8% of mortalities occurring in individuals aged 60 years and above (Table 1). COVID-19 cases and mortalities were observed in four waves, with increasing trends being observed across the study period. COVID-19 cases and deaths were lowest immediately after the first movement control order between March and April 2020. (Fig. 1a). Predicted all-cause mortality trends were observed to be bimodal, with peaks between December 2020-February 2021 and May–July 2021. Observed all-cause mortality trends declined across 2020 before an exponential increase in 2021. A reduction of between 9,798 and 26,522 deaths (reduction of 5.5–23.7%) was observed between predicted and observed mortalities in 2020. Observed all-cause mortalities exceeded predicted all-cause mortalities in 2021. An increase of 18,220 and 30,526 deaths (excess of 13.0–24.0%) was reported in 2021. The proportion of excess mortalities attributable to COVID-19 in 2021 ranged from 86.2% to 100%. Excess mortalities were largest between July and September 2021, corresponding to counts of 20,718 to 24,027 all-cause mortalities (excess of 46.4–58.1%) compared to predicted mortalities (Fig. 1b). Trends of COVID-19 cases and deaths corresponded to observed all-cause mortality trends in 2021.

**Table 1:** Characteristics of all mortalities over the study period (January 2016-December 2020).

	Total mortalities (n = 1,000,562)	Mortalities (January 2016–December 2019) (n = 676,146)	Mortalities (January 2020–December 2020) (n = 166,507)	Mortalities (January 2021–September 2021) (n = 157,909)	p-value
Age, mean (SD)	65.6 (18.6)	65.3 (19.1)	66.4 (17.8)	66.0 (17.1)	< 0.01^a^
**Age (%)**					< 0.01^b^
Less than 1*	17,979 (1.8)	13,625 (2.0)	2696 (1.6)	1658 (1.0)	
Less than 5	22,501 (2.2)	17,189 (2.5)	3320 (2.0)	1992 (1.3)	
6 to 14	6079 (0.6)	4704 (0.7)	803 (0.5)	572 (0.4)	
15 to 40	83,805 (8.4)	58,539 (8.7)	13,035 (7.8)	12,231 (7.7)	
41 to 59	219,350 (21.9)	146,516 (21.7)	36,254 (21.8)	36,580 (23.2)	
More than 60	668,827 (66.8)	449,198 (66.4)	113,095 (67.9)	106,534 (67.5)	
**Sex (%)**					< 0.01^b^
Male	575,248 (57.5)	388,850 (57.5)	96,322 (57.8)	90,076 (57.0)	
**States (%)**					< 0.01^b^
Johor	123,932 (12.4)	83,479 (12.3)	20,615 (12.4)	19,838 (12.6)	
Kedah	84,383 (8.4)	56,947 (8.4)	13,665 (8.2)	13,771 (8.7)	
Kelantan	65,485 (6.5)	44,717 (6.6)	10,919 (6.6)	9849 (6.2)	
Melaka	32,386 (3.2)	21,658 (3.2)	5440 (3.3)	5288 (3.3)	
Negeri Sembilan	42,271 (4.2)	28,376 (4.2)	6999 (4.2)	6896 (4.4)	
Pahang	52,733 (5.3)	36,550 (5.4)	8673 (5.2)	7510 (4.8)	
Perak	106,445 (10.6)	731,79 (10.8)	17,928 (10.8)	15,338 (9.7)	
Perlis	11,419 (1.1)	7924 (1.2)	1950 (1.2)	1545 (1.0)	
Pulau Pinang	64,305 (6.4)	43,544 (6.4)	10,689 (6.4)	10,072 (6.4)	
Sabah	78,726 (7.9)	54,986 (8.1)	14,629 (8.8)	9111 (5.8)	
Sarawak	79,275 (7.9)	53,543 (7.9)	14,071 (8.5)	11,661 (7.4)	
Selangor	162,071 (16.2)	107,408 (15.9)	5359 (15.2)	29,304 (18.6)	
Terengganu	40,861 (4.1)	28,324 (4.2)	6728 (4.0)	5809 (3.7)	
W.P. Kuala Lumpur	53,241 (5.3)	33,586 (5.0)	8318 (5.0)	11,337 (7.2)	
W.P. Labuan	1798 (0.2)	1107 (0.2)	314 (0.2)	377 (0.2)	
W.P. Putrajaya	1231 (0.1)	818 (0.1)	210 (0.1)	203 (0.1)	
**Road-traffic accident-related deaths (%)**					< 0.01^b^
Yes	23,701 (2.4)	18,563 (2.7)	3167 (1.9)	1971 (1.2)	

*SD* standard deviation.

*Under 1 mortality is a subset of under-5 mortalities.

^a^Significance was tested using a two-way t-test.

^b^significance was tested using a chi-square test.

**Figure 2:**
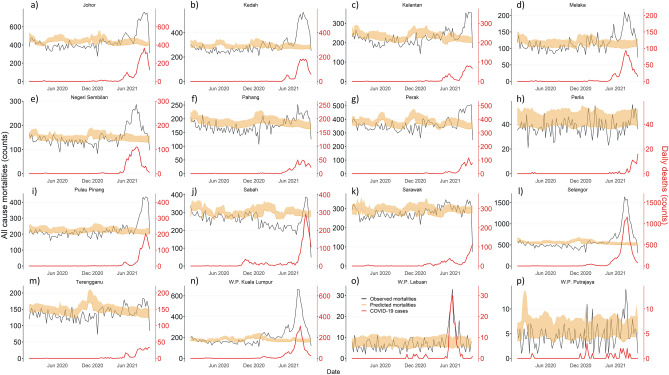
Predicted and Observed all-cause mortalities in Malaysia by states of (**a**) Johor, (**b**) Kedah, (**c**) Kelantan, (**d**) Melaka, (**e**) Negeri Sembilan, (**f**) Pahang, (**g**) Perak, (**h**) Perlis, (**i**) Pulau Pinang, (**j**) Sabah, (**k**) Sarawak, (**l**) Selangor, (**m**) Terengganu, (**n**) W.P Kuala Lumpur, (**o**) W.P. Labuan, and (**p**) W.P. Putrajaya.

The states of Johor, Kedah, Perak, Perlis, Pulau Pinang, Sabah, and Sarawak reflected the national bimodal trend of predicted mortalities. The states of Kelantan, Melaka, Negeri Sembilan, Pahang, and Terengganu were observed to report unimodal predicted all-cause mortality trends, whilst Selangor, Putrajaya, Kuala Lumpur and Labuan were observed to have a static trend. All states observed larger all-cause mortalities than the upper interval of predicted mortalities between July and September 2021 (Fig. 2). Excess counts were observed to be largest in the states of Terengganu (excess of between 2.2–19.5%), Pahang (excess of 10.2–22.4%), Perak (excess of 14.1–25.5%), Kelantan (excess of 17.6–28.8%), Melaka (excess of 23.4–37.4%), Negeri Sembilan (excess of 25.8–38.0%), Johor (excess of 28.0–36.6%), Pulau Pinang (excess of 29.3–39.4%), Kedah (excess of 35.5–43.3%), Selangor (excess of 47.6–53.6%), and Kuala Lumpur (excess of 53.3–61.3%) between July and September 2021. The proportion of excess mortalities attributable to COVID-19 in these states ranged from 51.6% to 100% (Supplementary Appendix 3).

**Figure 3:**
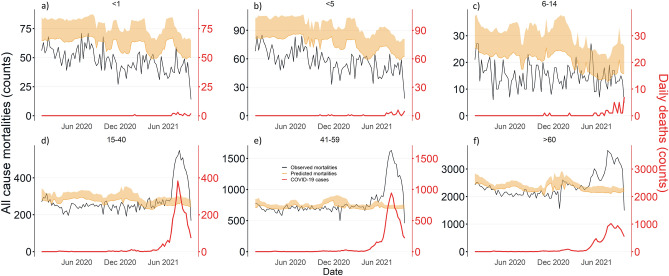
Predicted and observed all-cause mortalities in Malaysia by age groups of (**a**) Less than 1 year old, (**b**) Less than 5 years old, (**c**) 6–14 years old, (**d**) 15–40 years old, (**e**) 41–59 years old and (**f**) 60 years old and above.

Predicted mortalities in individuals above 40 years of age were observed to be bimodal, whilst younger age groups did not exhibit any particular trend. Observed all-cause mortalities were reported to exceed predicted mortalities in all age groups above 14 beyond July 2021 (Fig. 3). Excess counts were observed to be the largest in individuals aged ≥ 60 (excess of 27.8–33.7%), between 41 and 59 (excess of 41.7–45.9%), and between 15 and 40 (excess of 32.0–41.5%) between July and September 2021. The proportion of excess mortalities attributable to COVID-19 in these age groups ranged from 74.8% to more than 100% (Supplementary Appendix 3).

**Figure 4:**
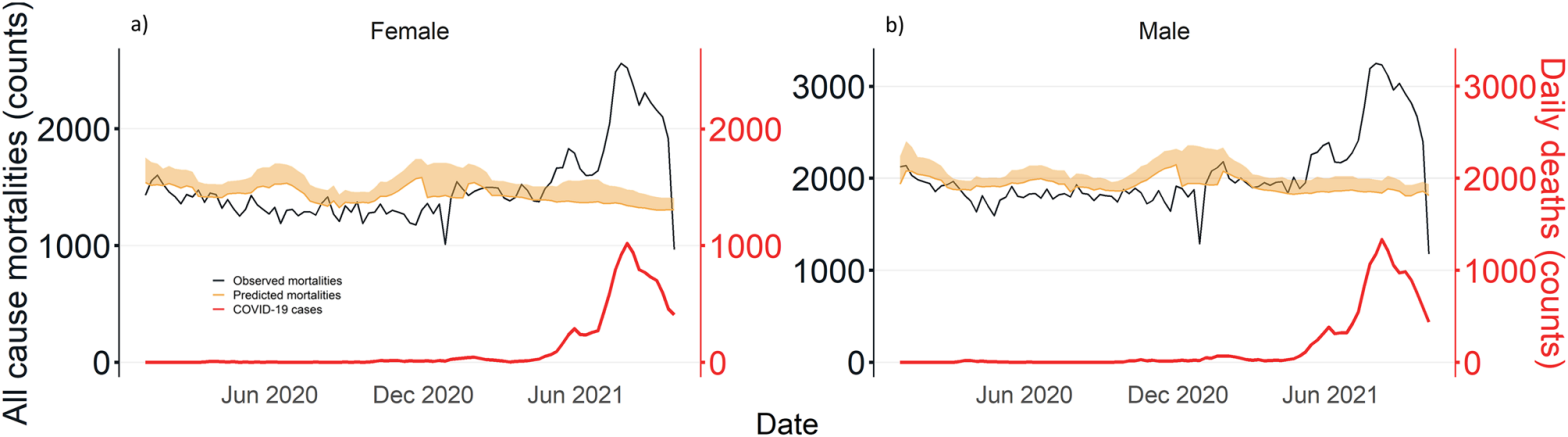
Predicted and observed all-cause mortalities in Malaysia by sex; (**a**) Female and (**b**) Male.

Predicted all-cause mortality trends in males and females correspond to national bimodal trends. Observed all-cause mortalities were reported to peak between July and September 2021. Observed all-cause mortalities are reported to exceed predicted mortalities beyond July 2021 (Fig. 4). Excess counts were the largest in males (excess of 30.5–35.0%) and females (excess of 33.3–39.1%) between July and September 2021. The proportion of excess mortalities attributable to COVID-19 in these age groups ranged from 82.5% to more than 100% (Supplementary Appendix 3).

**Figure 5:**
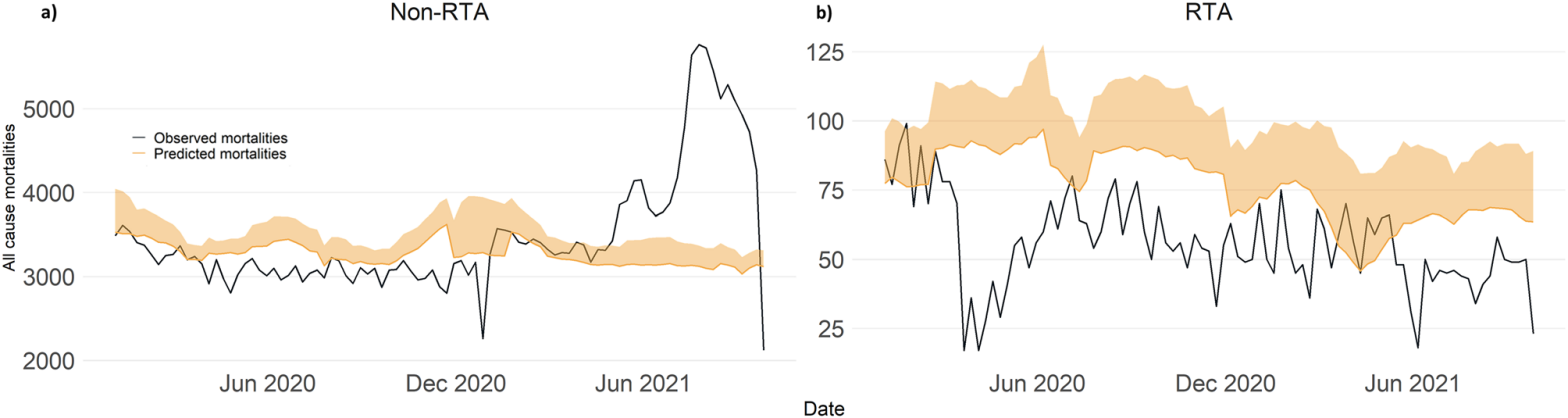
Predicted and observed all-cause mortalities in Malaysia by cause of death; (**a**) Non road-traffic accidents and (**b**) Road-traffic accidents.

Predicted non-RTA mortality trends corresponded to the bimodal national all-cause mortality trends. Predicted RTA trends were observed to decrease across time. Observed non-RTA mortalities were observed to peak between July and September 2021—consistent with trends of all-cause mortality at the national level (Fig. 5). Observed RTA mortalities were reported to be consistently lower than predicted across the study period by between 36.6 and 80.5%. The largest difference between predicted and observed mortalities was in April–June 2020 (reduction of 86.7 to 135.7%) (Supplementary Appendix Table 4). No major differences in trend were observed between overall estimations and stratification by ethnicity and nationality (Supplementary Appendix 5, 6, and 7). The Bayesian hierarchical estimates were off similar trend to the Farrington algorithm but with larger uncertainties and higher excess thresholds. Both models suggest no excess in 2020, and excess in 2021. Excess of 33.6–38.1% (Farrington) and 22.1–31.9% (Bayesian Hierarchical) is estimated for July–September 2021 (Supplementary Appendix 8).

## Discussion

Over the course of nearly 21 months of a global pandemic, Malaysia has observed an overall reduction in observed mortalities from predicted mortalities across 2020. There is almost no discernible change in all-cause mortality trends within Malaysia's early days of the COVID-19 pandemic. The findings here corroborate that local transmission was likely low in early 2020, as reported by the national surveillance system^38,39^. This trend was reversed in 2021, with large increases in excess all-cause mortalities, especially between July and September 2021. This trend corresponds to large increases in COVID-19 transmission observed during this period as the Delta variant swept across the country^8,10^. However, we found that a large proportion of these all-cause mortalities can be attributed to COVID-19.

Excess counts were almost non-existent and sometimes negative in Malaysia over 2020. A reduction in observed mortalities from predicted mortalities is not unique to Malaysia as several other countries such as Australia, New Zealand, and Mongolia also reported similar findings—that have been linked to the implementation of stringent lockdown and social-distancing measures^40,41^. In New Zealand, these reductions have been attributed to reduced influenza, pneumonia, RTA, occupational causes of death, air pollution, and post-surgical complications that were all reduced due to the lockdown^41^. In the short term, this may have negated the increase of mortalities from COVID-19 and potential acute collateral effects such as suicide from psychological distress, home injury death, and the effects of disruption in healthcare services.

Nonetheless, a reversal of this trend was observed in 2021—with excesses being observed as COVID-19 transmission increased, leading to potential undiagnosed COVID-19 deaths, acute collateral effects of an overwhelmed health system and the side effects of strong restrictive non-pharmaceutical interventions, as has been suggested in previous studies^42–45^. It has been reported that the COVID-19 pandemic has disrupted essential health services in 90% of countries^46^. Countries such as the United Kingdom have reported a substantial reduction in admission rates for acute coronary syndromes, with the fear of contracting COVID-19 infection cited as a significant reason^47^. The decrease in acute stroke management and admissions was similarly reported in many countries during this pandemic^48^. A preliminary survey by the Malaysia Stroke Council suggested that a decline in hospital presentation with acute stroke symptoms also occurred in Malaysia similarly due to a fear of being exposed to COVID-19^49^. Ischemic heart disease and stroke are the top causes of death in Malaysia, accounting for 23.0% of medically certified mortality in the country^50^. Nonetheless, the proportion of excess deaths attributable to these factors remains unknown and should be investigated in future research.

All-cause mortalities in individuals under the age of 15 appear to be on a downwards trend across the study period. We postulate that this notable decrease in infant, below-five, and 6–14 mortality trends may be due to a reduction in non-COVID-19 pneumonia mortality, the most common cause of mortality among Malaysians under the age of 15^33^. This reduction in non-COVID-19 pneumonia is potentially facilitated by a combination of reduced human–human interaction, increased use of masks, compliance to hand hygiene, and physical distancing among children and adolescents following the implementation of wide-scale public health and social measures. Reductions in pneumonia-associated mortalities within the context of COVID-19 were similarly reported in New Zealand and Japan^40,41,51,52^. Nonetheless, this association here remains conjecture and will require more work before a definitive answer is discovered.

We observed a substantial reduction in RTA mortalities, especially during the MCO. In Malaysia, road and transport-related accidents are the leading cause of death in individuals aged between 15 and 40^33,53,54^. Movement restriction measures such as the MCO in Malaysia led to a sharp decrease in human mobility and are speculated to be the reason for the acute decrease in mortality, especially in individuals in the 15–40 age group^55^. There is evidence complementary to our findings that lockdowns reduce accidental mortalities^56^.

Despite almost 21 months of being in a pandemic, there remains brevity of published tracking of excess mortality in the region. A literature search on the WHO COVID-19 database found one of 180 pre-publications on excess mortality from Southeast Asia^57^. A majority of these publications come from Europe, where robust national vital-statistics surveillance programs support dedicated excess mortality projects^17^. COVID-19 is a reminder of the importance of improving our national vital statistics surveillance and pooling resources in developing a regional system that will serve as a surveillance indicator for future pandemics, influenza, and other public health threats^58^.

The use of aberration detection mechanisms in the surveillance of excess mortalities here has several vital strengths. The dataset utilised here is large, with 21 months of data representing the effects of the COVID-19 pandemic. It is, as such likely to be a reliable representation of the Malaysian population during the COVID-19 pandemic. The methodology utilised here is robust and has been utilised within several large surveillance collaboratives^17^. The sensitivity analysis utilising a more complex local model produced smaller but similar trends with large excess mortalities during the Delta wave in Malaysia suggesting the Farrington algorithm remains a robust estimator of excess mortalities. Despite this, there were several limitations. Completion of the primary vaccine series in Malaysia was only 8.1% when the case fatality rate peaked at 2.2% on 4 July 2021. The vaccination rate reached 64% by 30 September 2021, with a case fatality rate of 0.8%^55^. While there could have been a potential effect from the vaccinations on excess mortalities, we did not consider the effects due to the unavailability of longer data periods. A total of 94.4–96.1% of all data were reported within the same month in January and February 2020. This dropped to 61–85% between March and July 2020 and increased to more than 90% after August 2020^59^. Reporting delay in the data is unlikely in 2020, but a possibility within 2021 as data up to September 2021 was extracted in October 2021. Nonetheless, based on previous trends, more than 90% of all reported deaths are likely to have been reported for all months except September 2021, as is also observed by the tapering of reporting in the last two weeks of analysis. The analysis did not correct for these reporting delays. Additionally, under-reporting was suspected to be possible in East Malaysia (Sabah, Sarawak, and Labuan). However, as this study analysed the trend of all-cause mortality, it is unlikely that this affected the analysis, as any underreporting effect is likely to occur at similar proportions across the entire study period. Nonetheless, exacerbations in under-reporting of mortalities due to COVID-19 cannot be ruled out here. Interpretation of the time trends is limited by specific causes of death, except for RTA, due to coding delays and coronial inquiries. It is also important to note that the low actual and predicted number of mortalities in Labuan and Putrajaya may result in imprecise estimates. We could not utilise small area estimation as we did not have access to more granular data. Future work will focus on improving estimations at more granular spatial dimensions. We are also unable to disentangle the extent of excess mortalities due to COVID and non-COVID causes amid the syndemic.

## Conclusion

We found no excess all-cause mortality for Malaysia in 2020. Significant increases in transmission have led to excess mortalities observed in 2021, especially between July and September 2021. Our findings highlight the importance of monitoring the trends in all-cause mortality and in identifying any excess in all-cause mortality in situations such as the current COVID-19 pandemic. All-cause mortalities remain a valuable adjunct to traditional surveillance mechanisms. Surveillance of mortality in the future can increase the capacity of health systems to detect aberrant events for more timely public health interventions. Current surveillance strategies should incorporate excess mortality surveillance, particularly within resource-scarce settings. It is also essential to keep in mind that the true burden of collateral mortalities remains hidden for now. Consistent tracking of mortalities must be continued as we continue to deal with COVID-19 whilst slowly coming to grips with its fallout.

## Supplementary Information

Supplementary Information.

## Data Availability

The COVID-19 datasets analysed during the current study are available in the Ministry of Health Malaysia COVID-19 repository (https://github.com/MoH-Malaysia/covid19-public). The all-cause mortality data that support the findings of this study are available from the Department of Statistics, Malaysia but restrictions apply to the availability of these data, which were used under license for the current study, and so are not publicly available. Data are however available upon reasonable request from, and with permission of, the Department of Statistics, Malaysia (mystats.sec@dosm.gov.my).
