# Supplementary material for: Estimating excess mortalities due to the COVID-19 pandemic in Malaysia between January 2020 and September 2021
Source: Sci Rep. 2023 Jan 3;13:86. doi: 10.1038/s41598-022-26927-z (PMC9807979; doi:10.1038/s41598-022-26927-z)
Supplement: Supplementary file 1 — Supplementary Information. [file 41598_2022_26927_MOESM1_ESM.docx]

Supplementary Appendix

Estimating excess mortalities in Malaysia between January 2020 and September 2021

Vivek Jason Jayaraj^1,2^, Diane Woei-Quan Chong^1,2^, Kim-Sui Wan^1,2^, Noran Naqiah Hairi^1^, Nirmala Bhoo-Pathy^1^, Sanjay Rampal^1^, Chiu-Wan Ng*^1^

^1^ Centre for Epidemiology and Evidence-based Practice, Department of Social and Preventive Medicine, Faculty of Medicine, University of Malaya, 50603 Kuala Lumpur, Malaysia

^2^ Ministry of Health Malaysia, Federal Government Administrative Centre, 62590 Putrajaya, Malaysia.

*Corresponding author:

Chiu-Wan, Ng, PhD

Centre for Epidemiology and Evidence-based Practice,

Department of Social and Preventive Medicine,

Faculty of Medicine, University of Malaya,

50603 Kuala Lumpur, Malaysia

Tel: +60.3.79674760

Email: chiuwan.ng@ummc.edu.my

Appendix 1 STL decomposition of a) Counts, b) Seasonality, c) Trends, and d) Noise associated with time series of all-cause mortalities in Malaysia between January 2020 and September 2021


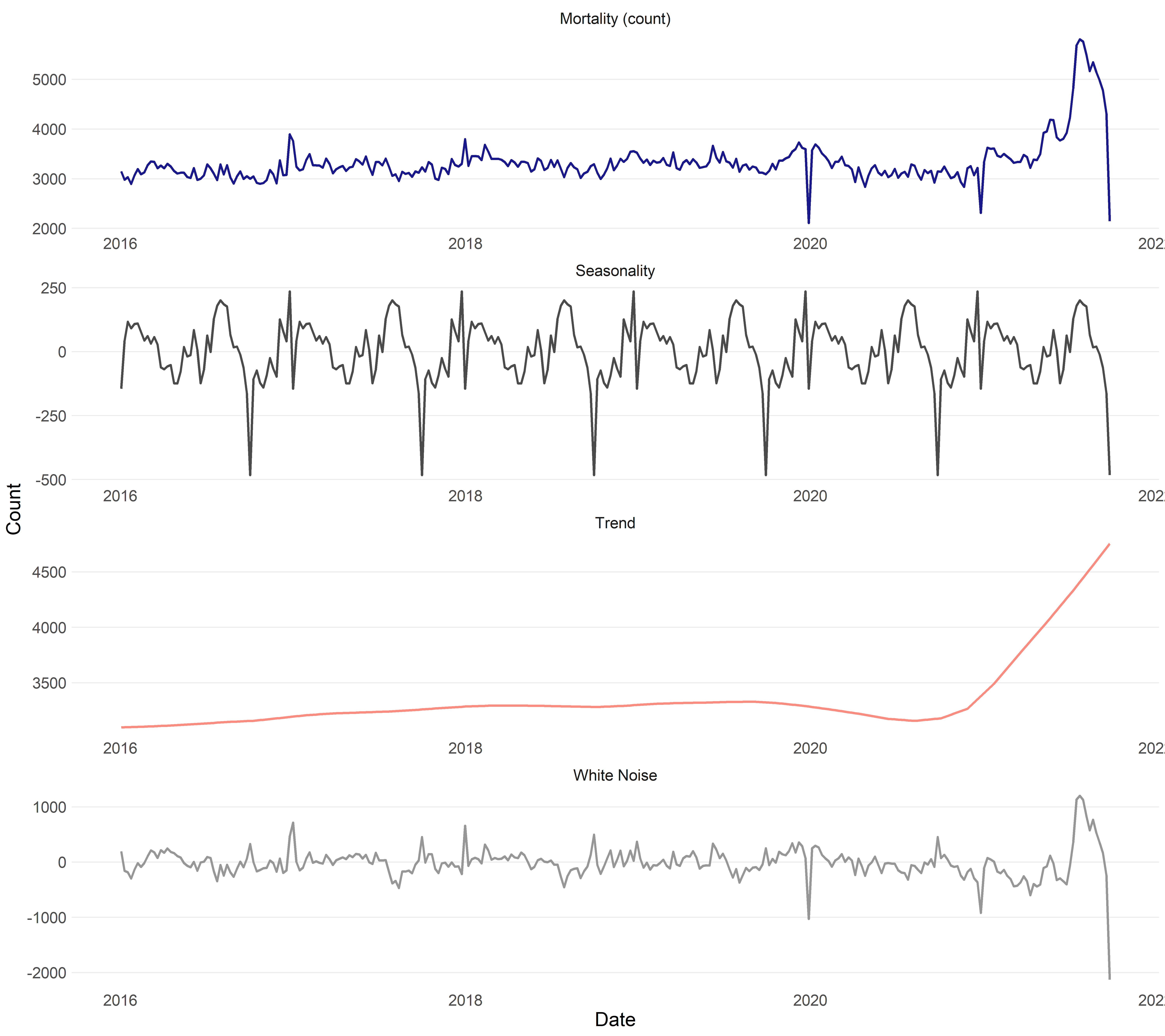


Appendix 2 Observed, predicted and COVID-19 associated mortalities between January 2020-September 2021

|  | Total | | | | | |  |
| --- | --- | --- | --- | --- | --- | --- | --- |
|  |  |  |  |  |  |  |  |
|  | Observed mortalities | Predicted mortalities (95% CI Upper limit) | Excess counts | Percentage change | COVID-19 Mortalities | Excess deaths attributable to COVID-19 |  |
| Age (years) |  |  |  |  |  |  |  |
| Less than 1* | 4307 | 5554 (7193) | -2886 , -1247 | -67 , -29 | 11 | -0·4, -0·9 |  |
| Less than 5 | 5250 | 6885 (8806) | -3556 , -1635 | -67·7 , -31·1 | 35 | -1, -2·1 |  |
| 6 to 14 | 1355 | 1837 (2779) | -1424 , -482 | -105·1 , -35·6 | 39 | -2·7, -8·1 |  |
| 15 to 40 | 25029 | 24289 (28126) | -3097 , 740 | -12·4 , 3 | 3168 | -102·3, 428·1 |  |
| 41 to 59 | 72220 | 64912 (71920) | 300 , 7308 | 0·4 , 10·1 | 9134 | 3044·7, 125 |  |
| More than 60 | 217511 | 206466 (226945) | -9434 , 11045 | -4·3 , 5·1 | 14673 | -155·5, 132·8 |  |
| States^a^ |  |  |  |  |  |  |  |
| Johor | 40068 | 37909 (43464) | -3396 , 2159 | -8·5 , 5·4 | 3421 | -100·7, 158·5 |  |
| Kedah | 27187 | 24992 (28898) | -1711 , 2195 | -6·3 , 8·1 | 1893 | -110·6, 86·2 |  |
| Kelantan | 20575 | 19732 (23162) | -2587 , 843 | -12·6 , 4·1 | 878 | -33·9, 104·2 |  |
| Melaka | 10654 | 9663 (11852) | -1198 , 991 | -11·2 , 9·3 | 865 | -72·2, 87·3 |  |
| Negeri Sembilan | 13765 | 12629 (15136) | -1371 , 1136 | -10 , 8·3 | 1198 | -87·4, 105·5 |  |
| Pahang | 16032 | 15964 (18597) | -2565 , 68 | -16 , 0·4 | 598 | -23·3, 879·4 |  |
| Perak | 32922 | 32322 (37209) | -4287 , 600 | -13 , 1·8 | 927 | -21·6, 154·5 |  |
| Perlis | 3465 | 3452 (4673) | -1208 , 13 | -34·9 , 0·4 | 79 | -6·5, 607·7 |  |
| Pulau Pinang | 20573 | 18957 (22247) | -1674 , 1616 | -8·1 , 7·9 | 1426 | -85·2, 88·2 |  |
| Sabah | 23498 | 25613 (29556) | -6058 , -2115 | -25·8 , -9 | 2292 | -37·8, -108·4 |  |
| Sarawak | 25452 | 24993 (29093) | -3641 , 459 | -14·3 , 1·8 | 941 | -25·8, 205 |  |
| Selangor | 54158 | 48319 (54592) | -434 , 5839 | -0·8 , 10·8 | 9446 | -2176·5, 161·8 |  |
| Terengganu | 12431 | 12519 (14973) | -2542 , -88 | -20·4 , -0·7 | 394 | -15·5, -447·7 |  |
| W·P· Kuala Lumpur | 19492 | 15241 (18167) | 1325 , 4251 | 6·8 , 21·8 | 2521 | 190·3, 59·3 |  |
| W·P· Labuan | 683 | 484 (936) | -253 , 199 | -37 , 29·2 | 149 | -58·9, 74·9 |  |
| W·P· Putrajaya | 410 | 372 (746) | -336 , 38 | -82 , 9·4 | 21 | -6·2, 55·3 |  |
| Sex |  |  |  |  |  |  |  |
| Female | 136683 | 129490 (142963) | -6280 , 7193 | -4·6 , 5·3 | 11569 | -184·2, 160·8 |  |
| Male | 184683 | 174198 (189755) | -5072 , 10485 | -2·7 , 5·7 | 15479 | -305·2, 147·6 |  |
| Notes: |  |  |  |  |  |  |  |
| A positive value is interpreted as excess mortality, whilst a negative value is interpreted as a reduction of observed mortalities from predicted mortalities· | | | | | | |  |
| *Age less than 1 is a subset of age less than 5- as such cumulative will be in excess of total counts, | | | | | | |  |

| Appendix 3 Observed, predicted and COVID-19 associated mortalities from three-monthly periods between January 2020-September 2021 | | | | | | | |
| --- | --- | --- | --- | --- | --- | --- | --- |
|  | | | | January-March 2020 | | | |
|  | Observed mortalities | Predicted mortalities (95% CI Upper limit) | Excess counts | | Percentage change | COVID-19 Mortalities | Excess deaths attributable to COVID-19 |
| Age (years) |  |  |  | |  |  |  |
| Less than 1 | 735 | 853 (1094) | -359 , -118 | | -48·9 , -16 | 0 | 0, 0 |
| Less than 5 | 942 | 1069 (1362) | -420 , -127 | | -44·5 , -13·5 | 0 | 0, 0 |
| 6 to 14 | 242 | 304 (436) | -194 , -62 | | -80·1 , -25·8 | 0 | 0, 0 |
| 15 to 40 | 3494 | 3546 (4095) | -601 , -52 | | -17·2 , -1·5 | 7 | -1·2, -13·5 |
| 41 to 59 | 9130 | 9292 (10354) | -1224 , -162 | | -13·4 , -1·8 | 14 | -1·1, -8·6 |
| More than 60 | 30723 | 31049 (34045) | -3322 , -326 | | -10·8 , -1·1 | 28 | -0·8, -8·6 |
| States^a^ |  |  |  | |  |  |  |
| Johor | 5592 | 5353 (6130) | -538 , 239 | | -9·6 , 4·3 | 13 | -2·4, 5·4 |
| Kedah | 3632 | 3614 (4210) | -578 , 18 | | -15·9 , 0·5 | 1 | -0·2, 5·6 |
| Kelantan | 3013 | 3012 (3515) | -502 , 1 | | -16·6 , 0 | 2 | -0·4, 200 |
| Melaka | 1416 | 1455 (1774) | -358 , -39 | | -25·3 , -2·7 | 2 | -0·6, -5·1 |
| Negeri Sembilan | 1893 | 1897 (2244) | -351 , -4 | | -18·5 , -0·2 | 2 | -0·6, -50 |
| Pahang | 2335 | 2442 (2846) | -511 , -107 | | -21·9 , -4·6 | 0 | 0, 0 |
| Perak | 4757 | 4683 (5373) | -616 , 74 | | -13 , 1·6 | 3 | -0·5, 4·1 |
| Perlis | 481 | 494 (681) | -200 , -13 | | -41·6 , -2·8 | 1 | -0·5, -7·7 |
| Pulau Pinang | 2782 | 2780 (3288) | -506 , 2 | | -18·2 , 0·1 | 1 | -0·2, 50 |
| Sabah | 3787 | 3722 (4377) | -590 , 65 | | -15·6 , 1·7 | 1 | -0·2, 1·5 |
| Sarawak | 3584 | 3552 (4278) | -694 , 32 | | -19·4 , 0·9 | 8 | -1·2, 25 |
| Selangor | 7040 | 7112 (7934) | -894 , -72 | | -12·7 , -1 | 7 | -0·8, -9·7 |
| Terengganu | 1782 | 1846 (2157) | -375 , -64 | | -21 , -3·6 | 0 | 0, 0 |
| W·P· Kuala Lumpur | 2304 | 2112 (2484) | -180 , 192 | | -7·8 , 8·3 | 7 | -3·9, 3·6 |
| W·P· Labuan | 76 | 72 (133) | -57 , 4 | | -75·5 , 5 | 0 | 0, 0 |
| W·P· Putrajaya | 57 | 60 (116) | -59 , -3 | | -103·3 , -4·5 | 1 | -1·7, -33·3 |
| Sex |  |  |  | |  |  |  |
| Female | 19011 | 19300 (21189) | -2178 , -289 | | -11·5 , -1·5 | 13 | -0·6, -4·5 |
| Male | 25520 | 25561 (27928) | -2408 , -41 | | -9·4 , -0·2 | 35 | -1·5, -85·4 |
| Notes: | | | | | | | |
| A positive value is interpreted as excess mortality, whilst a negative value is interpreted as a reduction of observed mortalities from predicted mortalities· | | | | | | | |
| *Age less than 1 is a subset of age less than 5- as such cumulative will be in excess of total counts | | | | | | | |
| ^a^ Imported cases (n=400) were left out of the analysis | | | | | | | |

| Appendix 3 Observed, predicted and COVID-19 associated mortalities from three-monthly periods between January 2020-September 2021 (continued) | | | | | | | |
| --- | --- | --- | --- | --- | --- | --- | --- |
|  | | | | April-June 2020 | | | |
|  | Observed mortalities | Predicted mortalities (95% CI Upper limit) | Excess counts | | Percentage change | COVID-19 Mortalities | Excess deaths attributable to COVID-19 |
| Age (years) |  |  |  | |  |  |  |
| Less than 1 | 676 | 849 (1076) | -400 , -173 | | -59·2 , -25·6 | 0 | 0, 0 |
| Less than 5 | 809 | 1084 (1360) | -551 , -275 | | -68·2 , -34 | 0 | 0, 0 |
| 6 to 14 | 176 | 292 (429) | -253 , -116 | | -143·8 , -65·8 | 0 | 0, 0 |
| 15 to 40 | 3048 | 3739 (4226) | -1178 , -691 | | -38·7 , -22·7 | 6 | -0·5, -0·9 |
| 41 to 59 | 8901 | 9241 (10182) | -1281 , -340 | | -14·4 , -3·8 | 13 | -1, -3·8 |
| More than 60 | 27307 | 30111 (32601) | -5294 , -2804 | | -19·4 , -10·3 | 54 | -1, -1·9 |
| States^a^ |  |  |  | |  |  |  |
| Johor | 4855 | 5711 (6485) | -1630 , -856 | | -33·6 , -17·6 | 7 | -0·4, -0·8 |
| Kedah | 3201 | 3727 (4244) | -1043 , -526 | | -32·6 , -16·4 | 0 | 0, 0 |
| Kelantan | 2527 | 2822 (3354) | -827 , -295 | | -32·7 , -11·7 | 1 | -0·1, -0·3 |
| Melaka | 1329 | 1359 (1639) | -310 , -30 | | -23·3 , -2·2 | 4 | -1·3, -13·3 |
| Negeri Sembilan | 1765 | 1806 (2143) | -378 , -41 | | -21·4 , -2·3 | 6 | -1·6, -14·6 |
| Pahang | 2185 | 2260 (2611) | -426 , -75 | | -19·5 , -3·4 | 7 | -1·6, -9·3 |
| Perak | 4403 | 4782 (5438) | -1035 , -379 | | -23·5 , -8·6 | 3 | -0·3, -0·8 |
| Perlis | 477 | 481 (642) | -165 , -4 | | -34·6 , -0·8 | 1 | -0·6, -25 |
| Pulau Pinang | 2483 | 2718 (3169) | -686 , -235 | | -27·6 , -9·5 | 0 | 0, 0 |
| Sabah | 3529 | 3646 (4155) | -626 , -117 | | -17·8 , -3·3 | 6 | -1, -5·1 |
| Sarawak | 3472 | 3602 (4122) | -650 , -130 | | -18·7 , -3·7 | 9 | -1·4, -6·9 |
| Selangor | 6228 | 6961 (7794) | -1566 , -733 | | -25·1 , -11·8 | 17 | -1·1, -2·3 |
| Terengganu | 1651 | 1767 (2086) | -435 , -116 | | -26·3 , -7 | 1 | -0·2, -0·9 |
| W·P· Kuala Lumpur | 2011 | 2175 (2561) | -550 , -164 | | -27·3 , -8·2 | 11 | -2, -6·7 |
| W·P· Labuan | 78 | 68 (144) | -66 , 10 | | -84·6 , 12·7 | 0 | 0, 0 |
| W·P· Putrajaya | 47 | 56 (114) | -67 , -9 | | -141·5 , -19·2 | 0 | 0, 0 |
| Sex |  |  |  | |  |  |  |
| Female | 17169 | 19200 (21032) | -3863 , -2031 | | -22·5 , -11·8 | 20 | -0·5, -1 |
| Male | 23072 | 25271 (26901) | -3829 , -2199 | | -16·6 , -9·5 | 53 | -1·4, -2·4 |
| Notes: | | | | | | | |
| A positive value is interpreted as excess mortality, whilst a negative value is interpreted as a reduction of observed mortalities from predicted mortalities· | | | | | | | |
| *Age less than 1 is a subset of age less than 5- as such cumulative will be in excess of total counts | | | | | | | |
| ^a^ Imported cases (n=400) were left out of the analysis | | | | | | | |

| Appendix *3* Observed, predicted and COVID-19 associated mortalities from three-monthly periods between January 2020-September 2021 (continued) | | | | | | | |
| --- | --- | --- | --- | --- | --- | --- | --- |
|  | | | | July-September 2020 | | | |
|  | Observed mortalities | Predicted mortalities (95% CI Upper limit) | Excess counts | | Percentage change | COVID-19 Mortalities | Excess deaths attributable to COVID-19 |
| Age (years) |  |  |  | |  |  |  |
| Less than 1 | 712 | 860 (1091) | -379 , -148 | | -53·2 , -20·9 | 0 | 0, 0 |
| Less than 5 | 845 | 1063 (1328) | -483 , -218 | | -57·2 , -25·8 | 0 | 0, 0 |
| 6 to 14 | 188 | 281 (423) | -235 , -93 | | -124·7 , -49·3 | 0 | 0, 0 |
| 15 to 40 | 3264 | 3571 (4097) | -833 , -307 | | -25·5 , -9·4 | 0 | 0, 0 |
| 41 to 59 | 9084 | 9145 (9870) | -786 , -61 | | -8·7 , -0·7 | 5 | -0·6, -8·2 |
| More than 60 | 27477 | 28757 (31119) | -3642 , -1280 | | -13·3 , -4·7 | 12 | -0·3, -0·9 |
| States^a^ |  |  |  | |  |  |  |
| Johor | 5118 | 5405 (6108) | -990 , -287 | | -19·3 , -5·6 | 1 | -0·1, -0·3 |
| Kedah | 3297 | 3520 (3982) | -685 , -223 | | -20·8 , -6·8 | 5 | -0·7, -2·2 |
| Kelantan | 2714 | 2648 (3050) | -336 , 66 | | -12·4 , 2·4 | 0 | 0, 0 |
| Melaka | 1264 | 1354 (1660) | -396 , -90 | | -31·4 , -7·1 | 1 | -0·3, -1·1 |
| Negeri Sembilan | 1714 | 1718 (2062) | -348 , -4 | | -20·3 , -0·2 | 0 | 0, 0 |
| Pahang | 2092 | 2203 (2542) | -450 , -111 | | -21·5 , -5·3 | 0 | 0, 0 |
| Perak | 4345 | 4400 (5071) | -726 , -55 | | -16·7 , -1·3 | 0 | 0, 0 |
| Perlis | 490 | 509 (682) | -192 , -19 | | -39·1 , -4 | 0 | 0, 0 |
| Pulau Pinang | 2704 | 2626 (3077) | -373 , 78 | | -13·8 , 2·9 | 1 | -0·3, 1·3 |
| Sabah | 3556 | 3540 (4008) | -452 , 16 | | -12·7 , 0·4 | 7 | -1·5, 43·8 |
| Sarawak | 3480 | 3541 (4028) | -548 , -61 | | -15·7 , -1·7 | 2 | -0·4, -3·3 |
| Selangor | 6273 | 6887 (7693) | -1420 , -614 | | -22·6 , -9·8 | 0 | 0, 0 |
| Terengganu | 1650 | 1685 (2035) | -385 , -35 | | -23·3 , -2·1 | 0 | 0, 0 |
| W·P· Kuala Lumpur | 2034 | 2165 (2621) | -587 , -131 | | -28·9 , -6·4 | 0 | 0, 0 |
| W·P· Labuan | 72 | 69 (131) | -59 , 3 | | -82 , 4 | 0 | 0, 0 |
| W·P· Putrajaya | 55 | 51 (105) | -50 , 4 | | -91·6 , 6·9 | 0 | 0, 0 |
| Sex |  |  |  | |  |  |  |
| Female | 17021 | 17908 (19466) | -2445 , -887 | | -14·4 , -5·2 | 5 | -0·2, -0·6 |
| Male | 23837 | 24571 (26282) | -2445 , -734 | | -10·3 , -3·1 | 12 | -0·5, -1·6 |
| Notes: | | | | | | | |
| A positive value is interpreted as excess mortality, whilst a negative value is interpreted as a reduction of observed mortalities from predicted mortalities· | | | | | | | |
| *Age less than 1 is a subset of age less than 5- as such cumulative will be in excess of total counts | | | | | | | |
| ^a^ Imported cases (n=400) were left out of the analysis | | | | | | | |

| Appendix *3* Observed, predicted and COVID-19 associated mortalities from three-monthly periods between January 2020-September 2021 (continued) | | | | | | | |
| --- | --- | --- | --- | --- | --- | --- | --- |
|  | | | | October-December 2020 | | | |
|  | Observed mortalities | Predicted mortalities (95% CI Upper limit) | Excess counts | | Percentage change | COVID-19 Mortalities | Excess deaths attributable to COVID-19 |
| Age (years) |  |  |  | |  |  |  |
| Less than 1 | 573 | 763 (993) | -420 , -190 | | -73·4 , -33·1 | 0 | 0, 0 |
| Less than 5 | 724 | 999 (1249) | -525 , -275 | | -72·6 , -38 | 3 | -0·6, -1·1 |
| 6 to 14 | 197 | 296 (433) | -236 , -99 | | -119·8 , -50·1 | 2 | -0·8, -2 |
| 15 to 40 | 3229 | 3574 (4149) | -920 , -345 | | -28·5 , -10·7 | 28 | -3, -8·1 |
| 41 to 59 | 9139 | 9885 (11241) | -2102 , -746 | | -23 , -8·2 | 103 | -4·9, -13·8 |
| More than 60 | 27588 | 29614 (33602) | -6014 , -2026 | | -21·8 , -7·3 | 242 | -4, -11·9 |
| States^a^ |  |  |  | |  |  |  |
| Johor | 5050 | 5509 (6392) | -1342 , -459 | | -26·6 , -9·1 | 16 | -1·2, -3·5 |
| Kedah | 3535 | 3625 (4274) | -739 , -90 | | -20·9 , -2·6 | 6 | -0·8, -6·7 |
| Kelantan | 2665 | 2946 (3452) | -787 , -281 | | -29·5 , -10·5 | 6 | -0·8, -2·1 |
| Melaka | 1431 | 1389 (1748) | -317 , 42 | | -22·1 , 3 | 1 | -0·3, 2·4 |
| Negeri Sembilan | 1627 | 1849 (2269) | -642 , -222 | | -39·4 , -13·6 | 5 | -0·8, -2·3 |
| Pahang | 2061 | 2283 (2690) | -629 , -222 | | -30·5 , -10·8 | 0 | 0, 0 |
| Perak | 4423 | 4889 (5746) | -1323 , -466 | | -29·9 , -10·5 | 10 | -0·8, -2·1 |
| Perlis | 502 | 489 (673) | -171 , 13 | | -34·1 , 2·5 | 0 | 0, 0 |
| Pulau Pinang | 2720 | 2768 (3250) | -530 , -48 | | -19·5 , -1·8 | 8 | -1·5, -16·7 |
| Sabah | 3757 | 3603 (4240) | -483 , 154 | | -12·9 , 4·1 | 261 | -54, 169·5 |
| Sarawak | 3535 | 3603 (4260) | -725 , -68 | | -20·5 , -1·9 | 2 | -0·3, -2·9 |
| Selangor | 5818 | 7290 (8432) | -2614 , -1472 | | -44·9 , -25·3 | 43 | -1·6, -2·9 |
| Terengganu | 1645 | 2001 (2468) | -823 , -356 | | -50 , -21·6 | 0 | 0, 0 |
| W·P· Kuala Lumpur | 1969 | 2401 (2884) | -915 , -432 | | -46·5 , -21·9 | 10 | -1·1, -2·3 |
| W·P· Labuan | 88 | 66 (128) | -40 , 22 | | -45·5 , 25·3 | 8 | -20, 36·4 |
| W·P· Putrajaya | 51 | 52 (106) | -55 , -1 | | -107·3 , -1·5 | 2 | -3·6, -200 |
| Sex |  |  |  | |  |  |  |
| Female | 16984 | 19006 (21402) | -4418 , -2022 | | -26 , -11·9 | 144 | -3·3, -7·1 |
| Male | 23893 | 25488 (28829) | -4936 , -1595 | | -20·7 , -6·7 | 234 | -4·7, -14·7 |
| Notes: | | | | | | | |
| A positive value is interpreted as excess mortality, whilst a negative value is interpreted as a reduction of observed mortalities from predicted mortalities· | | | | | | | |
| *Age less than 1 is a subset of age less than 5- as such cumulative will be in excess of total counts | | | | | | | |
| ^a^ Imported cases (n=400) were left out of the analysis | | | | | | | |

| Appendix *3* Observed, predicted and COVID-19 associated mortalities from three-monthly periods between January 2020-September 2021 (continued) | | | | | | | |
| --- | --- | --- | --- | --- | --- | --- | --- |
|  | | | | January-March 2021 | | | |
|  | Observed mortalities | Predicted mortalities (95% CI Upper limit) | Excess counts | | Percentage change | COVID-19 Mortalities | Excess deaths attributable to COVID-19 |
| Age (years) |  |  |  | |  |  |  |
| Less than 1 | 586 | 789 (1043) | -457 , -203 | | -77·9 , -34·7 | 1 | -0·2, -0·5 |
| Less than 5 | 689 | 966 (1264) | -575 , -277 | | -83·5 , -40·2 | 2 | -0·3, -0·7 |
| 6 to 14 | 203 | 266 (403) | -200 , -63 | | -98·4 , -31 | 1 | -0·5, -1·6 |
| 15 to 40 | 3142 | 3392 (3975) | -833 , -250 | | -26·5 , -8 | 52 | -6·2, -20·8 |
| 41 to 59 | 9416 | 9125 (10337) | -921 , 291 | | -9·8 , 3·1 | 186 | -20·2, 63·9 |
| More than 60 | 30871 | 30917 (34357) | -3486 , -46 | | -11·3 , -0·1 | 600 | -17·2, -1304·3 |
| States^a^ |  |  |  | |  |  |  |
| Johor | 5549 | 5484 (6359) | -810 , 65 | | -14·6 , 1·2 | 79 | -9·8, 121·5 |
| Kedah | 3700 | 3575 (4202) | -502 , 125 | | -13·6 , 3·4 | 14 | -2·8, 11·2 |
| Kelantan | 2905 | 2957 (3472) | -567 , -52 | | -19·5 , -1·8 | 15 | -2·6, -28·8 |
| Melaka | 1521 | 1416 (1753) | -232 , 105 | | -15·3 , 6·9 | 16 | -6·9, 15·2 |
| Negeri Sembilan | 1859 | 1875 (2241) | -382 , -16 | | -20·5 , -0·9 | 20 | -5·2, -125 |
| Pahang | 2291 | 2370 (2810) | -519 , -79 | | -22·7 , -3·4 | 15 | -2·9, -19 |
| Perak | 4747 | 4638 (5359) | -612 , 109 | | -12·9 , 2·3 | 28 | -4·6, 25·7 |
| Perlis | 522 | 493 (679) | -157 , 29 | | -30·1 , 5·5 | 0 | 0, 0 |
| Pulau Pinang | 2765 | 2773 (3275) | -510 , -8 | | -18·4 , -0·3 | 12 | -2·4, -150 |
| Sabah | 2922 | 3841 (4535) | -1613 , -919 | | -55·2 , -31·5 | 123 | -7·6, -13·4 |
| Sarawak | 3672 | 3517 (4259) | -587 , 155 | | -16 , 4·2 | 92 | -15·7, 59·4 |
| Selangor | 7119 | 6967 (7930) | -811 , 152 | | -11·4 , 2·1 | 303 | -37·4, 199·3 |
| Terengganu | 1860 | 1831 (2172) | -312 , 29 | | -16·8 , 1·5 | 12 | -3·8, 41·4 |
| W·P· Kuala Lumpur | 2748 | 2230 (2655) | 93 , 518 | | 3·4 , 18·9 | 103 | 110·8, 19·9 |
| W·P· Labuan | 83 | 72 (134) | -51 , 11 | | -60·9 , 12·9 | 4 | -7·8, 36·4 |
| W·P· Putrajaya | 58 | 49 (98) | -40 , 9 | | -69·6 , 15·6 | 5 | -12·5, 55·6 |
| Sex |  |  |  | |  |  |  |
| Female | 18791 | 18828 (20962) | -2171 , -37 | | -11·6 , -0·2 | 295 | -13·6, -797·3 |
| Male | 25530 | 25261 (28095) | -2565 , 269 | | -10 , 1·1 | 546 | -21·3, 203 |
| Notes: | | | | | | | |
| A positive value is interpreted as excess mortality, whilst a negative value is interpreted as a reduction of observed mortalities from predicted mortalities· | | | | | | | |
| *Age less than 1 is a subset of age less than 5- as such cumulative will be in excess of total counts | | | | | | | |
| ^a^ Imported cases (n=400) were left out of the analysis | | | | | | | |

| Appendix *3* Observed, predicted and COVID-19 associated mortalities from three-monthly periods between January 2020-September 2021 (continued) | | | | | | | |
| --- | --- | --- | --- | --- | --- | --- | --- |
|  | | | | April-June 2021 | | | |
|  | Observed mortalities | Predicted mortalities (95% CI Upper limit) | Excess counts | | Percentage change | COVID-19 Mortalities | Excess deaths attributable to COVID-19 |
| Age (years) |  |  |  | |  |  |  |
| Less than 1 | 541 | 720 (949) | -408 , -179 | | -75·4 , -33 | 0 | 0, 0 |
| Less than 5 | 665 | 855 (1134) | -469 , -190 | | -70·5 , -28·5 | 1 | -0·2, -0·5 |
| 6 to 14 | 188 | 200 (326) | -138 , -12 | | -73·2 , -6·1 | 3 | -2·2, -25 |
| 15 to 40 | 3467 | 3179 (3761) | -294 , 288 | | -8·5 , 8·3 | 286 | -97·3, 99·3 |
| 41 to 59 | 10393 | 9157 (10161) | 232 , 1236 | | 2·2 , 11·9 | 1099 | 473·7, 88·9 |
| More than 60 | 33526 | 28078 (30806) | 2720 , 5448 | | 8·1 , 16·3 | 2991 | 110, 54·9 |
| States^a^ |  |  |  | |  |  |  |
| Johor | 6020 | 5203 (6036) | -16 , 817 | | -0·3 , 13·6 | 486 | -3037·5, 59·5 |
| Kedah | 3961 | 3469 (4046) | -85 , 492 | | -2·1 , 12·4 | 226 | -265·9, 45·9 |
| Kelantan | 3213 | 2691 (3246) | -33 , 522 | | -1 , 16·2 | 232 | -703, 44·4 |
| Melaka | 1625 | 1351 (1637) | -12 , 274 | | -0·8 , 16·9 | 137 | -1141·7, 50 |
| Negeri Sembilan | 2274 | 1771 (2129) | 145 , 503 | | 6·4 , 22·1 | 399 | 275·2, 79·3 |
| Pahang | 2430 | 2241 (2594) | -164 , 189 | | -6·7 , 7·8 | 99 | -60·4, 52·4 |
| Perak | 4701 | 4538 (5164) | -463 , 163 | | -9·8 , 3·5 | 89 | -19·2, 54·6 |
| Perlis | 458 | 480 (640) | -182 , -22 | | -39·7 , -4·7 | 6 | -3·3, -27·3 |
| Pulau Pinang | 2970 | 2663 (3121) | -151 , 307 | | -5·1 , 10·3 | 99 | -65·6, 32·2 |
| Sabah | 2804 | 3623 (4127) | -1323 , -819 | | -47·2 , -29·2 | 158 | -11·9, -19·3 |
| Sarawak | 4119 | 3577 (4082) | 37 , 542 | | 0·9 , 13·1 | 309 | 835·1, 57 |
| Selangor | 8259 | 6635 (7510) | 749 , 1624 | | 9·1 , 19·7 | 1491 | 199·1, 91·8 |
| Terengganu | 1897 | 1736 (2048) | -151 , 161 | | -8 , 8·5 | 64 | -42·4, 39·8 |
| W·P· Kuala Lumpur | 3261 | 2095 (2471) | 790 , 1166 | | 24·2 , 35·8 | 466 | 59, 40 |
| W·P· Labuan | 192 | 70 (143) | 49 , 122 | | 25·7 , 63·5 | 114 | 232·7, 93·4 |
| W·P· Putrajaya | 55 | 51 (100) | -45 , 4 | | -82·6 , 7·9 | 5 | -11·1, 125 |
| Sex |  |  |  | |  |  |  |
| Female | 20634 | 17940 (19959) | 675 , 2694 | | 3·3 , 13·1 | 1819 | 269·5, 67·5 |
| Male | 27605 | 24032 (26042) | 1563 , 3573 | | 5·7 , 12·9 | 2561 | 163·9, 71·7 |
| Notes: | | | | | | | |
| A positive value is interpreted as excess mortality, whilst a negative value is interpreted as a reduction of observed mortalities from predicted mortalities· | | | | | | | |
| *Age less than 1 is a subset of age less than 5- as such cumulative will be in excess of total counts | | | | | | | |
| ^a^ Imported cases (n=400) were left out of the analysis | | | | | | | |

| Appendix *3* Observed, predicted and COVID-19 associated mortalities from three-monthly periods between January 2020-September 2021 (continued) | | | | | | | |
| --- | --- | --- | --- | --- | --- | --- | --- |
|  | | | | July-September 2021 | | | |
|  | Observed mortalities | Predicted mortalities (95% CI Upper limit) | Excess counts | | Percentage change | COVID-19 Mortalities | Excess deaths attributable to COVID-19 |
| Age (years) |  |  |  | |  |  |  |
| Less than 1 | 531 | 721 (947) | -416 , -190 | | -78·3 , -35·7 | 10 | -2·4, -5·3 |
| Less than 5 | 638 | 848 (1108) | -470 , -210 | | -73·7 , -33 | 28 | -6, -13·3 |
| 6 to 14 | 181 | 199 (330) | -149 , -18 | | -82·4 , -10·2 | 31 | -20·8, -172·2 |
| 15 to 40 | 5622 | 3288 (3823) | 1799 , 2334 | | 32 , 41·5 | 2767 | 153·8, 118·6 |
| 41 to 59 | 16771 | 9067 (9773) | 6998 , 7704 | | 41·7 , 45·9 | 7662 | 109·5, 99·5 |
| More than 60 | 42137 | 27940 (30415) | 11722 , 14197 | | 27·8 , 33·7 | 10615 | 90·6, 74·8 |
| States^a^ |  |  |  | |  |  |  |
| Johor | 8269 | 5245 (5955) | 2314 , 3024 | | 28 , 36·6 | 2797 | 120·9, 92·5 |
| Kedah | 6110 | 3462 (3940) | 2170 , 2648 | | 35·5 , 43·3 | 1621 | 74·7, 61·2 |
| Kelantan | 3731 | 2657 (3075) | 656 , 1074 | | 17·6 , 28·8 | 602 | 91·8, 56·1 |
| Melaka | 2142 | 1340 (1641) | 501 , 802 | | 23·4 , 37·4 | 699 | 139·5, 87·2 |
| Negeri Sembilan | 2763 | 1712 (2049) | 714 , 1051 | | 25·8 , 38 | 767 | 107·4, 73 |
| Pahang | 2789 | 2165 (2504) | 285 , 624 | | 10·2 , 22·4 | 471 | 165·3, 75·5 |
| Perak | 5890 | 4391 (5058) | 832 , 1499 | | 14·1 , 25·5 | 774 | 93, 51·6 |
| Perlis | 565 | 506 (676) | -111 , 59 | | -19·7 , 10·5 | 69 | -62·2, 116·9 |
| Pulau Pinang | 4337 | 2629 (3067) | 1270 , 1708 | | 29·3 , 39·4 | 1278 | 100·6, 74·8 |
| Sabah | 3385 | 3639 (4113) | -728 , -254 | | -21·5 , -7·5 | 1720 | -236·3, -677·2 |
| Sarawak | 3870 | 3601 (4065) | -195 , 269 | | -5 , 7 | 482 | -247·2, 179·2 |
| Selangor | 13926 | 6467 (7300) | 6626 , 7459 | | 47·6 , 53·6 | 7566 | 114·2, 101·4 |
| Terengganu | 2052 | 1653 (2007) | 45 , 399 | | 2·2 , 19·5 | 309 | 686·7, 77·4 |
| W·P· Kuala Lumpur | 5328 | 2064 (2490) | 2838 , 3264 | | 53·3 , 61·3 | 1918 | 67·6, 58·8 |
| W·P· Labuan | 102 | 66 (123) | -21 , 36 | | -20·7 , 35 | 22 | -104·8, 61·1 |
| W·P· Putrajaya | 90 | 53 (107) | -17 , 37 | | -18·7 , 40·6 | 8 | -47·1, 21·6 |
| Sex |  |  |  | |  |  |  |
| Female | 28408 | 17308 (18953) | 9455 , 11100 | | 33·3 , 39·1 | 9158 | 96·9, 82·5 |
| Male | 36941 | 24014 (25678) | 11263 , 12927 | | 30·5 , 35 | 11945 | 106·1, 92·4 |
|  |  |  |  | |  |  |  |
| Notes: | | | | | | | |
| A positive value is interpreted as excess mortality, whilst a negative value is interpreted as a reduction of observed mortalities from predicted mortalities· | | | | | | | |
| *Age less than 1 is a subset of age less than 5- as such cumulative will be in excess of total counts | | | | | | | |
| ^a^ Imported cases (n=400) were left out of the analysis | | | | | | | |

Appendix 4 Sensitivity analysis of observed and predicted mortalities associated with road-traffic and non-road traffic accidents between January 2020- September 2021

|  | Observed mortalities | Predicted mortalities (95% CI Upper limit) | Excess counts | Percentage change |
| --- | --- | --- | --- | --- |
| RTA |  |  |  |  |
| Cumulative | 5069 | 6922 (9149) | -4080 , -1853 | -80·5 , -36·6 |
| January-March 2020 | 968 | 1086 (1369) | -401 , -118 | -41·4 , -12·2 |
| April-June 2020 | 623 | 1163 (1468) | -845 , -540 | -135·7 , -86·7 |
| July-September 2020 | 864 | 1125 (1435) | -571 , -261 | -66·1 , -30·2 |
| October-December 2020 | 712 | 1024 (1335) | -623 , -312 | -87·5 , -43·9 |
| January-March 2021 | 722 | 910 (1257) | -535 , -188 | -74·2 , -26 |
| April-June 2021 | 651 | 751 (1130) | -479 , -100 | -73·6 , -15·3 |
| July-September 2021 | 598 | 862 (1154) | -556 , -264 | -93 , -44·2 |
| Non-RTA |  |  |  |  |
| Cumulative | 316297 | 297117 (323458) | -7161 , 19180 | -2·3 , 6·1 |
| January-March 2020 | 43563 | 44076 (48067) | -4504 , -513 | -10·3 , -1·2 |
| April-June 2020 | 39618 | 43455 (46462) | -6844 , -3837 | -17·3 , -9·7 |
| July-September 2020 | 39994 | 41666 (44607) | -4613 , -1672 | -11·5 , -4·2 |
| October-December 2020 | 40165 | 43480 (48740) | -8575 , -3315 | -21·3 , -8·3 |
| January-March 2021 | 43599 | 43068 (47677) | -4078 , 531 | -9·4 , 1·2 |
| April-June 2021 | 47588 | 40874 (44411) | 3177 , 6714 | 6·7 , 14·1 |
| July-September 2021 | 64751 | 40497 (43494) | 21257 , 24254 | 32·8 , 37·5 |

Appendix 5 Sensitivity analysis of observed and predicted mortalities all-cause mortalities between January 2020-September 2021 by nationality and ethnicity

|  | Total | | | |  |
| --- | --- | --- | --- | --- | --- |
|  |  |  |  |  |  |
|  | Observed mortalities | Predicted mortalities (95% CI Upper limit) | Excess counts | Percentage change |  |
| Ethnicity |  |  |  |  |  |
| Malay | 168441 | 158374 (173713) | -5272 , 10067 | -3.1 , 6 |  |
| Other indigenous | 31937 | 32521 (37048) | -5111 , -584 | -16 , -1.8 |  |
| Chinese | 85886 | 80758 (89924) | -4038 , 5128 | -4.7 , 6 |  |
| Indian | 28434 | 26232 (30392) | -1958 , 2202 | -6.9 , 7.7 |  |
| Others | 6660 | 6131 (7718) | -1058 , 529 | -15.9 , 7.9 |  |
| Nationality |  |  |  |  |  |
| Malaysian | 315412 | 298490 (324785) | -9373 , 16922 | -3 , 5.4 |  |
| Non-Malaysian | 5946 | 5618 (7234) | -1288 , 328 | -21.7 , 5.5 |  |
| Notes: |  |  |  |  |  |
| A positive value is interpreted as an excess mortality whilst a negative value is interpreted as a reduction of observed mortalities from predicted mortalities. | | | | |  |

| Appendix 5 Sensitivity analysis of observed and predicted mortalities all-cause mortalities between January 2020-September 2021 by nationality and ethnicity (cotinued) | | | | | | | | |
| --- | --- | --- | --- | --- | --- | --- | --- | --- |
|  | January-March 2020 | | | | April-June 2020 | | | |
|  | Observed mortalities | Predicted mortalities (95% CI Upper limit) | Excess counts | Percentage change | Observed mortalities | Predicted mortalities (95% CI Upper limit) | Excess counts | Percentage change |
| Ethnicity | 12143 | 11842 (13128) | -985 , 301 | -8.1 , 2.5 | 10792 | 11356 (12522) | -1730 , -564 | -16 , -5.2 |
| Malay | 3888 | 3733 (4288) | -400 , 155 | -10.3 , 4 | 3312 | 3969 (4503) | -1191 , -657 | -35.9 , -19.8 |
| Other indigenous | 22977 | 23322 (25534) | -2557 , -345 | -11.1 , -1.5 | 20856 | 23446 (25276) | -4420 , -2590 | -21.2 , -12.4 |
| Chinese | 4671 | 4916 (5728) | -1057 , -245 | -22.6 , -5.2 | 4466 | 4613 (5145) | -679 , -147 | -15.2 , -3.3 |
| Indian | 852 | 888 (1116) | -264 , -36 | -31 , -4.2 | 815 | 900 (1119) | -304 , -85 | -37.3 , -10.4 |
| Others |  |  |  |  |  |  |  |  |
| Nationality | 43696 | 44271 (48308) | -4612 , -575 | -10.6 , -1.3 | 39520 | 43685 (46572) | -7052 , -4165 | -17.8 , -10.5 |
| Malaysian | 835 | 861 (1088) | -253 , -26 | -30.3 , -3.1 | 721 | 851 (1078) | -357 , -130 | -49.6 , -18.1 |
| Non-Malaysian | 12143 | 11842 (13128) | -985 , 301 | -8.1 , 2.5 | 10792 | 11356 (12522) | -1730 , -564 | -16 , -5.2 |
| Notes:  A positive value is interpreted as an excess mortality whilst a negative value is interpreted as a reduction of observed mortalities from predicted mortalities. | | | | | | | | |

| Appendix 5 Sensitivity analysis of observed and predicted mortalities all-cause mortalities between January 2020-September 2021 by nationality and ethnicity (cotinued) | | | | | | | | |
| --- | --- | --- | --- | --- | --- | --- | --- | --- |
|  | July-September 2020 | | | | October-December 2020 | | | |
|  | Observed mortalities | Predicted mortalities (95% CI Upper limit) | Excess counts | Percentage change | Observed mortalities | Predicted mortalities (95% CI Upper limit) | Excess counts | Percentage change |
| Ethnicity | 11039 | 11418 (12450) | -1411 , -379 | -12.8 , -3.4 | 11181 | 11983 (13878) | -2697 , -802 | -24.1 , -7.2 |
| Malay | 3325 | 3731 (4332) | -1007 , -406 | -30.3 , -12.2 | 3294 | 3927 (4612) | -1318 , -633 | -40 , -19.2 |
| Other indigenous | 21122 | 21680 (23523) | -2401 , -558 | -11.4 , -2.6 | 20893 | 23562 (26412) | -5519 , -2669 | -26.4 , -12.8 |
| Chinese | 4551 | 4523 (5066) | -515 , 28 | -11.3 , 0.6 | 4755 | 4522 (5253) | -498 , 233 | -10.5 , 4.9 |
| Indian | 821 | 867 (1085) | -264 , -46 | -32.1 , -5.5 | 754 | 887 (1134) | -380 , -133 | -50.4 , -17.6 |
| Others |  |  |  |  |  |  |  |  |
| Nationality | 40146 | 41923 (44837) | -4691 , -1777 | -11.7 , -4.4 | 40236 | 43744 (48988) | -8752 , -3508 | -21.8 , -8.7 |
| Malaysian | 712 | 811 (1051) | -339 , -99 | -47.6 , -13.9 | 641 | 806 (1049) | -408 , -165 | -63.7 , -25.7 |
| Non-Malaysian | 11039 | 11418 (12450) | -1411 , -379 | -12.8 , -3.4 | 11181 | 11983 (13878) | -2697 , -802 | -24.1 , -7.2 |
| Notes:  A positive value is interpreted as an excess mortality whilst a negative value is interpreted as a reduction of observed mortalities from predicted mortalities. | | | | | | | | |

| Appendix 5 Sensitivity analysis of observed and predicted mortalities all-cause mortalities between January 2020-September 2021 by nationality and ethnicity (cotinued) | | | | | | | | |
| --- | --- | --- | --- | --- | --- | --- | --- | --- |
|  | January-March 2021 | | | | April-June 2021 | | | |
|  | Observed mortalities | Predicted mortalities (95% CI Upper limit) | Excess counts | Percentage change | Observed mortalities | Predicted mortalities (95% CI Upper limit) | Excess counts | Percentage change |
| Ethnicity | 11902 | 11866 (13369) | -1467 , 36 | -12.3 , 0.3 | 12923 | 11076 (12282) | 641 , 1847 | 5 , 14.3 |
| Malay | 3931 | 3763 (4341) | -410 , 168 | -10.4 , 4.3 | 4259 | 3586 (4197) | 62 , 673 | 1.5 , 15.8 |
| Other indigenous | 23402 | 22886 (25413) | -2011 , 516 | -8.6 , 2.2 | 25590 | 21999 (24206) | 1384 , 3591 | 5.4 , 14 |
| Chinese | 4275 | 4676 (5543) | -1268 , -401 | -29.7 , -9.4 | 4514 | 4623 (5139) | -625 , -109 | -13.8 , -2.4 |
| Indian | 811 | 879 (1112) | -301 , -68 | -37.1 , -8.4 | 953 | 862 (1083) | -130 , 91 | -13.6 , 9.6 |
| Others |  |  |  |  |  |  |  |  |
| Nationality | 43577 | 43285 (47949) | -4372 , 292 | -10 , 0.7 | 47400 | 41131 (44697) | 2703 , 6269 | 5.7 , 13.2 |
| Malaysian | 744 | 814 (1041) | -297 , -70 | -40 , -9.5 | 839 | 757 (980) | -141 , 82 | -16.8 , 9.8 |
| Non-Malaysian | 11902 | 11866 (13369) | -1467 , 36 | -12.3 , 0.3 | 12923 | 11076 (12282) | 641 , 1847 | 5 , 14.3 |
| Notes:  A positive value is interpreted as an excess mortality whilst a negative value is interpreted as a reduction of observed mortalities from predicted mortalities. | | | | | | | | |

| Appendix *5* Sensitivity analysis of observed and predicted mortalities all-cause mortalities between January 2020-September 2021 by nationality and ethnicity *(cotinued)* | | | | | |
| --- | --- | --- | --- | --- | --- |
|  | July-September 2021 | | | | |
|  | Observed mortalities | Predicted mortalities (95% CI Upper limit) | Excess counts | Percentage change | |
| Ethnicity |  |  |  |  | |
| Malay | 35173 | 21480 (23349) | 11824 , 13693 | 33.6 , 38.9 | |
| Other indigenous | 5027 | 4648 (5175) | -148 , 379 | -2.9 , 7.5 | |
| Chinese | 16727 | 11217 (12294) | 4433 , 5510 | 26.5 , 32.9 | |
| Indian | 6718 | 3522 (4119) | 2599 , 3196 | 38.7 , 47.6 | |
| Others | 1704 | 849 (1069) | 635 , 855 | 37.3 , 50.2 | |
| Nationality |  |  |  |  | |
| Malaysian | 63840 | 40452 (43434) | 20406 , 23388 | 32 , 36.6 | |
| Non-Malaysian | 1509 | 719 (946) | 563 , 790 | 37.3 , 52.4 | |
| Notes: | | | | |  |
| A positive value is interpreted as an excess mortality whilst a negative value is interpreted as a reduction of observed mortalities from predicted mortalities. | | | | |  |


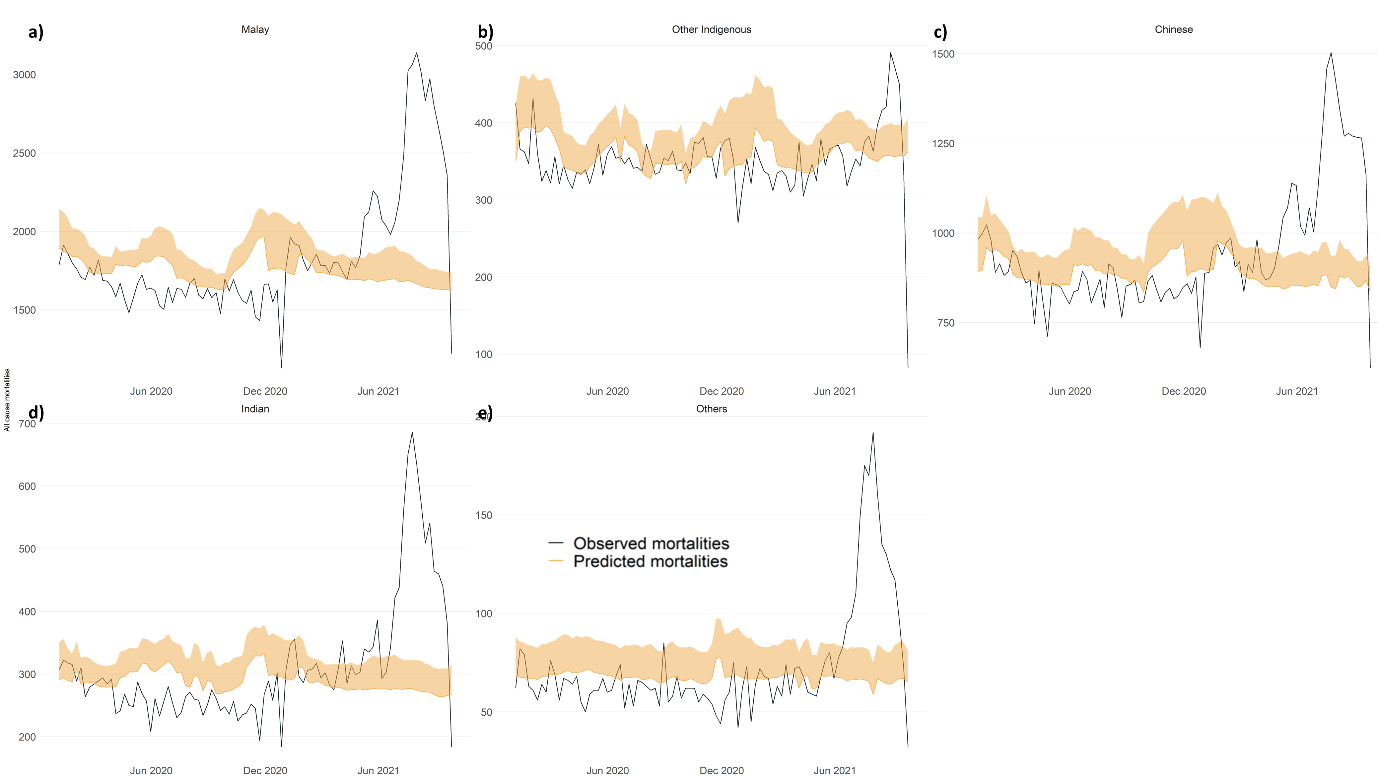


Appendix 6 Predicted and observed all-cause mortalities in Malaysia by age groups of a) Malay, b) Other Indigenous, c) Chinese, d) Indians, and e) Others


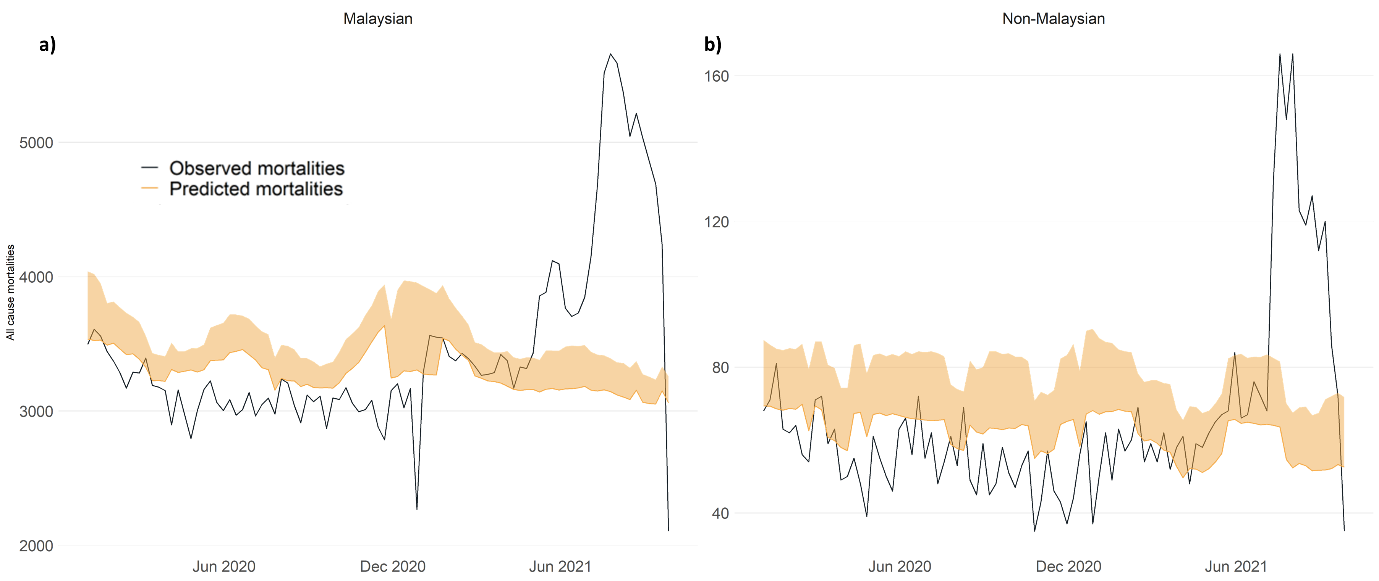


Appendix 7 Predicted and observed all-cause mortalities in Malaysia by nationality; a) Malaysians and b) Non-Malaysians

| Appendix 8: Comparison of the Farrington approach to the Bayesian hierarchical approach cumulatively across the study period, by years and over three month periods | | | | |
| --- | --- | --- | --- | --- |
|  | % change in Excess counts | | Proportion attributable to COVID-19 | |
|  | Farrington model | INLA model | Farrington model | INLA model |
| Cumulative | -2.4, 5.7 | -14.1, 0.2 | -346.6, 147.6 | -59.5, 3970.3 |
| 2020 | -17.2, -8.2 | -26.8, -10.9 | -1.9, -4 | -1.2, -3 |
| 2021 | 12.6, 19.8 | -1.2, 11.5 | 131.7, 83.8 | -1382.6, 144 |
| January- March 2020 | -10.4, -2.3 | -14.9, -0.5 | -1.6, -7.1 | -1.1, -35.5 |
| April-June 2020 | -18.2, -10.8 | -27.3, -11.3 | -0.8, -1.4 | -0.6, -1.3 |
| July-September 2020 | -12.9, -5.6 | -26.6, -10.7 | -0.4, -0.9 | -0.2, -0.5 |
| October-December 2020 | -27, -13.8 | -37.8, -20.6 | -3.5, -6.8 | -2.5, -4.6 |
| January- March 2021 | -8.8, 1.6 | -22.6, -7.2 | -21, 112.3 | -8.2, -25.7 |
| April-June 2021 | 5.8, 13.4 | -10.9, 3 | 166.1, 72.3 | -88.7, 318.4 |
| July-September 2021 | 33.6, 38.1 | 22.1, 31.9 | 97.7, 86 | 148.7, 102.9 |
